# Using the GOCE star trackers for validating the calibration of its accelerometers

**DOI:** 10.1007/s00190-017-1097-8

**Published:** 2017-12-08

**Authors:** P. N. A. M. Visser

**Affiliations:** Faculty of Aerospace Engineering, Delft University of Technology, Kluyverweg 1, 2629 HS Delft, The Netherlands

**Keywords:** GOCE, Gradiometer, Accelerometer, Star tracker, Calibration, Bias, Bias drift, Scale factor

## Abstract

A method for validating the calibration parameters of the six accelerometers on board the Gravity field and steady-state Ocean Circulation Explorer (GOCE) from star tracker observations that was originally tested by an end-to-end simulation, has been updated and applied to real data from GOCE. It is shown that the method provides estimates of scale factors for all three axes of the six GOCE accelerometers that are consistent at a level significantly better than 0.01 compared to the a priori calibrated value of 1. In addition, relative accelerometer biases and drift terms were estimated consistent with values obtained by precise orbit determination, where the first GOCE accelerometer served as reference. The calibration results clearly reveal the different behavior of the sensitive and less-sensitive accelerometer axes.

## Introduction

The Gravity field and steady-state Ocean Circulation Explorer (GOCE) is the first European Space Agency (ESA) earth explorer, launched on March 11, 2009 (Floberghagen et al. 2011; Drinkwater et al. 2007). The primary objective of the GOCE mission is to obtain a model for the mean Earth’s gravity field with an accuracy of better than 1 mgal for gravity anomalies and 1 cm for the geoid at a spatial resolution of 100 km or below. In order to meet this objective, the GOCE satellite is equipped with a number of instruments, including a gradiometer consisting of an orthogonal triad of three pairs of accelerometers, two high-precision dual-frequency Global Positioning System (GPS) receivers, three star trackers, and ion engines for flying drag-free. A prerequisite for GOCE’s success is a high-quality, high-precision calibration and validation of its accelerometers. Up to now, several methods have been proposed, including in-flight calibration (Frommknecht et al. 2011), use of star sensor data (Rispens and Bouman 2009; Siemes et al. 2012), and comparison with terrestrial gravimetry (Gruber et al. 2011). All methods have shown the high quality of the calibration of the GOCE official level-1b gravity gradient product (Frommknecht et al. 2011) in terms of scale factors for the measurement bandwidth (0.005–0.1 Hz). Since these methods in general only consider the signal in the measurement bandwidth (e.g., through bandpass filtering), they are not able to provide values for the accelerometer biases. In Visser et al. (2016) it is shown that this can be achieved by precise orbit determination, where GPS-based kinematic time series of GOCE positions are used to estimate accelerometer biases in a dynamic orbit fit.

**Fig. 1:**
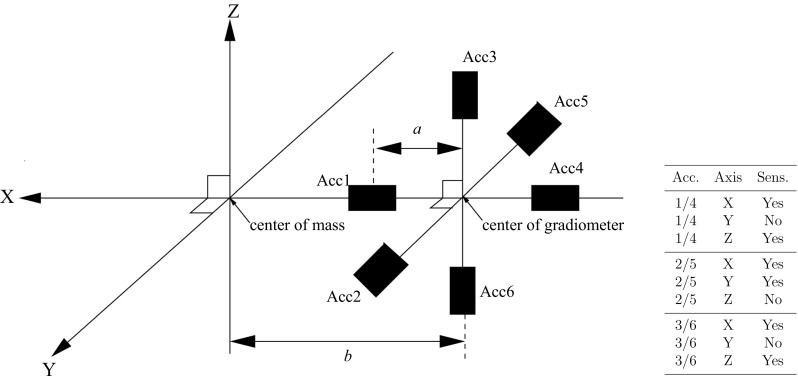
Configuration and naming convention of the 3 orthogonal pairs of accelerometers that form together the GOCE gravity gradiometer. The offset of the accelerometers is indicated by *a*, where *a* is equal to half the arm length for the associated axis. This arm length is equal to either 50.0 or 51.4 cm (Cesare and Catastini 2005). The offset of the center of the gradiometer with respect to the center of mass of the satellite is indicated by *b* (taken equal to zero for this study). The sensitive and less sensitive axes are indicated as well (Visser et al. 2016)

Already in e.g., ESA (1999), it is stated that the scale factor in the measurement bandwidth needs to be known with an accuracy of $10^{-5}$. To this aim, a procedure was designed and implemented that makes use of on-board shaking (Frommknecht et al. 2011). It has to be noted that the associated scale factors have been applied when generating the level-1b data that are used for testing the calibration method outlined in this paper. The expected value for the scale factors for the level-1b data is thus equal to 1.

An alternative method for estimating biases and scale factors was developed before the actual launch of GOCE and tested with data from an end-to-end simulator, which included full models of all instruments and their integration on the GOCE satellite (Visser 2008). This method allows the estimation of scale factors for all accelerometers and all axes, but not with the quality of the baseline method in Frommknecht et al. (2011). However, also relative accelerometer biases and drifts can be estimated, where one of the accelerometers needs to be defined as reference accelerometer. Although not all biases can be estimated, it will be shown that these relative biases can be compared with those published in Visser et al. (2016). The biases and drifts obtained by the method in Visser et al. (2016) are needed for using the accelerometer data for *e.g.,* thermospheric density and winds analysis. The results based on the star trackers provide an independent means of partly validating the bias and drift values in Visser et al. (2016). It is thus not expected that the calibration of the accelerometers by the method outlined in this paper leads to more precise scale factors and as such are not to be applied for gravity field retrieval. Values for the accelerometer biases and drifts are required for using the GOCE GPS satellite-to-satellite tracking (SST) data for retrieval of the long-wavelength part of the gravity field for GOCE-only models (Pail et al. 2011).

An important objective of this paper is to show that the method proposed in Visser (2008) confirms the high quality of the GOCE level-1b gravity gradient data in terms of accelerometer scale factors and also validates the accelerometer biases and drifts estimated by precise orbit determination (Visser et al. 2016). The remainder of this paper is organized as follows. The data set of GOCE observations that was used for obtaining estimates of accelerometer calibration parameters is described in Sect. 2. A recap of the method originally proposed in Visser (2008) is given in Sect. 3, where a few modifications based on experience with real data are addressed. Results are presented in Sect. 4 and the paper is completed with a summary, conclusions and recommendations in Sect. 5.

## Observations

For the method outlined in this paper, use is made of GOCE accelerometer and star tracker observations, kindly provided as level-1b data products through the asociated ESA web portal (http://eo-virtual-archive1.esa.int). The accelerometer observations are provided as time series with nominally a 1-s time step in the gradiometer reference frame (GRF). The orientation of this GRF in the J2000 reference frame is provided by quaternions derived from star tracker observations, which have a nominal time step of 0.5 s. It is assumed that the star tracker observations are properly calibrated. In Frommknecht et al. (2011), it is stated that misalignments between star sensor and gradiometer reference frames are sufficiently well known by manufacturing and verification on ground. The results described in this paper (Sect. 4) are based on selected days covering the period November 1, 2009–October 20, 2013, *i.e.,*the full GOCE operational period.

The six accelerometers of the gradiometer are schematically displayed in Fig. 1 (taken from Visser et al. 2016). The locations of the accelerometers are indicated along the *X*, *Y* and *Z* axes of the GRF. These axes are predominantly aligned with the along-track (or flight), cross-track and radial (or height) direction, respectively. Each accelerometer has two ultra-sensitive axes and one less-sensitive axis. It will be shown in the remainder of this paper that the calibration procedure outlined in this paper confirms the difference in sensitivity of the different accelerometer axes.

GOCE is equipped with three star camera head units (Frommknecht et al. 2011), where each camera head has a different orientation. With this arrangement it never happens that all three heads are blinded at the same time by the Sun and/or Moon. The different orientation of the camera heads means that so-called mounting matrices are used to derive the associated quaternions in the GRF. These mounting matrices are also provided as part of the level-1b products. Each camera head unit provides two very precise and one less precise orientation angle. The level-1b products include nominally the quaternions from two camera head units. It will be outlined in Sect. 3 that therefore always two time series are used together to estimate three precise orientation angles. Because of alternating possible blinding of star trackers by the Sun and/or Moon, also alternatingly observations of different pairs of star tracker camera observations are provided in the level-1b products. It was found that for 254 days no change occurred in the used pair of star tracker camera units. In order to avoid small jumps in orientation angles caused by *e.g.,* small errors in the mounting matrices, only these days were selected (“Appendix A”). As will be shown in Sect. 3, the orientation angles will be differentiated in time in order to obtain angular rates and accelerations. Possible jumps will then lead to large discontinuities in these rates and accelerations. It will be shown, however, that the observations of 254 days form together a sufficiently big data set to show the capabilities of the method. These 254 days include 31, 107, 59, 33 and 24 days in the years 2009, 2010, 2011, 2012 and 2013, respectively. The first day is November 7, 2009, and the last day October 20, 2013, which is close to the beginning and end of the GOCE operational mission phase, respectively. In the course of the GOCE operational mission, the number of switches between star camera head units increases due to the drift of the right ascension of ascending node being not perfectly synchronized with the rotation of the Earth around the Sun. This causes the GOCE orbit to slowly drift away from a dawn–dusk orbit. At the beginning of the operational phase, the local time of an ascending node passage was about 18:13, whereas this drifted to about 19:34 at the end of the operational mission.

## Methodology

The calibration of the accelerometers by the star tracker observation relies on prior knowledge of the Earth’s gravity field and on the observation or derivation of angular rates and accelerations. The observation equations are addressed first in Sect. 3.1, after which special attention is paid to the determination of angular rates and accelerations from the star tracker observations in Sect. 3.2.

### Observation equations

The observation equations that connect the accelerometer calibration parameters with the star tracker observations are based on the assumption that each GOCE accelerometer is affected by the same non-gravitational acceleration (Eq. (6) in Visser 2008). The accelerometer observations can be affected by misalignment errors and cross-coupling scale factor terms. Upper bounds were estimated for the misalignment error using Eq. (13) in Visser (2008) and were found to be significantly below 0.01 rad. For reference, the requirement for the combined effect of misalignments of, and couplings between, the accelerometer axes is smaller than $1.3 \times 10^{-4}$ rad (Cesare and Catastini 2005). It is therefore assumed that cross-coupling scale factor terms are absent. This leads to the following observation equation (Eq. (10) in Visser 2008):

1 $$\begin{aligned}&{} \mathbf{{S}}_i^{-1} ( \mathbf{{a}}_{\mathrm {obs},i} - \mathbf{{b}}_i -\mathbf{{\epsilon }}_i ) - ( {\varvec{\Gamma }} + \mathbf {R} ) \mathbf{{x}}_i \nonumber \\&\quad = \mathbf{{S}}_j^{-1} ( \mathbf{{a}}_{\mathrm {obs},j} - \mathbf{{b}}_j -\mathbf{{\epsilon }}_j ) - ( {\varvec{\Gamma }} + \mathbf {R} ) \mathbf{{x}}_j ~. \end{aligned}$$

The accelerations observed by the *i*th accelerometer ($i=1,\ldots ,6$) are represented by $\mathbf{{a}}_{\mathrm {obs},i}$, whereas the accelerometer biases, and the observation errors for the three accelerometer axes are represented by ${\mathbf{{b}}_i}^T=(b_{i,x},b_{i,y},b_{i,z})$ and ${\mathbf{{\epsilon }}_i}^T=(\epsilon _{i,x},\epsilon _{i,y},\epsilon _{i,z})$ (with *x*, *y*, *z* denoting the axes in the gradiometer reference frame (GRF)). The biases are assumed to drift linearly in time, *i.e.,*

2 $$\begin{aligned} \mathbf{b}_i ~=~ \mathbf{b}_{i,0} + \mathbf{b}_{i,t} t \end{aligned}$$

where $\mathbf{b}_{i,0}$ and $\mathbf{b}_{i,t}$ represent the biases at the starting epoch and the bias drifts. Time is represented by *t*. In *e.g.,* Visser et al. (2016) it was found that a linear model is capable of representing the long-term GOCE accelerometer bias behavior to within 1 nm/s$^2$ for the highest fidelity estimates along the GRF *X* axis.

The (diagonal) matrix of accelerometer scale factors $S_{i,k}$ ($k=x,y,z$) is represented by $\mathbf{S}_i$:

3 $$\begin{aligned} \mathbf{S}_i ~=~ \left( \begin{array}{ccc} S_{i,x} &{} 0 &{} 0 \\ 0 &{} S_{i,y} &{} 0 \\ 0 &{} 0 &{} S_{i,z} \\ \end{array} \right) \end{aligned}$$

Each accelerometer experiences different accelerations due to the local gravity gradient (${\varvec{\Gamma }}$) and due to rotational effects (angular accelerations and centrifugal terms $\mathbf{{R}}$). First of all, the location of each individual accelerometer has to be defined (represented by $\mathbf{{x}}_i$):

4 $$\begin{aligned} \mathbf{{x_1}}^T ~=~ (o_x+{L_x}/2,o_y,o_z) \nonumber \\ \mathbf{{x_2}}^T ~=~ (o_x,o_y+{L_y}/2,o_z) \nonumber \\ \mathbf{{x_3}}^T ~=~ (o_x,o_y,o_z+{L_z}/2) \nonumber \\ \mathbf{{x_4}}^T ~=~ (o_x-{L_x}/2,o_y,o_z) \nonumber \\ \mathbf{{x_5}}^T ~=~ (o_x,o_y-{L_y}/2,o_z) \nonumber \\ \mathbf{{x_6}}^T ~=~ (o_x,o_y,o_z-{L_z}/2) \end{aligned}$$

where $L_x,L_y,L_z$ are the gradiometer arm lengths along the GRF *X*, *Y* and *Z* axes (approximately 50 cm for each axis) and $\mathbf{{o}}^T=(o_x,o_y,o_z)$ represents the offset of the center of the gradiometer instrument with respect to the satellite center of mass (negligible).

${{\varvec{\Gamma }}}$ is the gravity gradient matrix containing the second-order derivatives of the gravitational field potential $\Gamma _{kl}$ ($k,l~=~x,y,z$) at the satellite location:

5 $$\begin{aligned} {\varvec{\Gamma }} = \left( \begin{array}{rrr} \Gamma _{xx} &{} \Gamma _{xy} &{} \Gamma _{xz} \\ \Gamma _{yx} &{} \Gamma _{yy} &{} \Gamma _{yz} \\ \Gamma _{zx} &{} \Gamma _{zy} &{} \Gamma _{zz} \\ \end{array} \right) \end{aligned}$$

The gravity gradients are computed using an a priori gravity field model (Sect. 4.2). The matrix with rotational terms $\mathbf{{R}}$ is written as (Rummel 1986):

6 $$\begin{aligned}&{} \mathbf{{R}}= \left( \begin{array}{ccc} r_{xx} &{} r_{xy} &{} r_{xz} \\ r_{yx} &{} r_{yy} &{} r_{yz} \\ r_{zx} &{} r_{zy} &{} r_{zz} \\ \end{array} \right) \nonumber \\&\quad =\left( \begin{array}{ccc} - {\omega _y}^2 - {\omega _z}^2 &{} \omega _y \omega _x &{} \omega _z \omega _x \\ \omega _x \omega _y &{} - {\omega _x}^2 - {\omega _z}^2 &{} \omega _z \omega _y \\ \omega _x \omega _z &{} \omega _y \omega _z &{} - {\omega _x}^2 - {\omega _y}^2 \\ \end{array} \right) \nonumber \\&\qquad + \left( \begin{array}{ccc} 0 &{} -\dot{\omega }_z &{} \dot{\omega }_y \\ \dot{\omega }_z &{} 0 &{} -\dot{\omega }_x \\ -\dot{\omega }_y &{} \dot{\omega }_x &{} 0 \\ \end{array} \right) \end{aligned}$$

where $\omega _k$ and $\dot{\omega }_k$ represent the angular rotation rates ${{\varvec{\omega }}}^T=(\omega _x,\omega _y,\omega _z)$ and the angular accelerations ${{\dot{\varvec{\omega }}}}^T=(\dot{\omega }_x,\dot{\omega }_y,\dot{\omega }_z)$. The elements of the matrix with rotational terms can be derived from the star tracker observations by single and double differentiation in time of observed orientation angles (see Sect. 3.2 below).

As outlined in Visser (2008), the observation equations are solved by the unweighted least-squares method. Looking at Eq. (1), observation noise and errors enter through both star tracker and accelerometer observations in the observation equations. The accelerometer observation noise and errors also enter in the partial derivatives of the observation equations to the estimated scale factors. The latter thus also affects the design matrix. This suggests it might be interesting to look at *e.g.,* total least-squares methods (Markovsky and Huffel 2007), because both dependent and independent variables are affected for this calibration method. For the accelerometers, the requirements indicate flat noise for the measurement bandwidth. Outside the measurement bandwidth, the noise increases with 1 / *f* at low frequencies and $f^2$ at high frequencies, with *f* representing the frequency (ESA 1999). The latter is the reason for applying bandpass filtering for other calibration methods that aim at estimating the accelerometer scale factors. However, this bandpass filtering destroys the observability of accelerometer bias and bias drifts. In Stummer et al. (2011), white noise is assumed for the star-tracker-derived orientation angles. This would lead to colored noise spectra where the amplitude is proportional with *f* and $f^2$ for rotation rates and accelerations, respectively.

It can thus not be claimed that the simple unweighted least-squares solver used in this paper leads to the best possible values for the estimated parameters. However, as explained above, several error sources with different character play a role making it not straightforward to design an alternative observation weighting scheme such that also the accelerometer biases and drifts can still be estimated. Moreover, as stated in Sect. 1, the primary objective of this paper is to show that the method as proposed in Visser (2008) has the capability to provide good estimates of accelerometer calibration parameters. It will be shown in Sect. 4 that this is indeed the case.

### Angular rotation rate and accelerations

For each star tracker, first and second time derivatives of the rotation angles are obtained in the star tracker reference frame (SRF) by using a moving time window of 50-s width (Sect. 4.1) over the time series of these angles and fitting second-order polynomials (as was also done in Visser 2008):

7 $$\begin{aligned} \phi _i ~=~ a_0 + a_1 t + \frac{1}{2} a_2 t^2 \end{aligned}$$

where $\phi _i$ represents the rotation angle with *i* denoting the associated SRF axis, *t* represents time, the coefficient $a_0$ represents the orientation angle at $t=0$ for the fitted polynomial, and $a_1$ and $a_2$ represent the angular rotation rate and angular acceleration. For each star tracker, the rotation angles around the $X_\mathrm{SRF}$ and $Y_\mathrm{SRF}$ axes are the most precise: the noise level for rotations around the $Z_\mathrm{SRF}$ bore sight axis is typically an order of magnitude larger.

If two differently oriented star trackers are observing simultaneously, two pairs of precise observations around the different $X_\mathrm{SRF}$ and $Y_\mathrm{SRF}$ axes can be used to derive precise rotation angles, rotation rates and angular accelerations around all three axes of the GRF. This is done by unweighted least-squares estimation (as stated above in *e.g.,* Stummer et al. (2011) white noise is assumed for the star-tracker-derived orientation angles). Please note that the combination of the star tracker observations is different from the method adopted in *e.g.,* Stummer et al. (2011), which is based on Wiener filtering in the spectral domain, whereas the method outlined above is based on a straightforward combination in the time domain. Please also note that for each star tracker thus only the two precise orientation angles are used, *i.e.,* four precise rotation angles of two differently oriented star trackers are used to derive the absolute 3-dimensional orientation in space. The observation equation is:

8 $$\begin{aligned} {o_s}_i ~=~ {r_s}_{ix} \alpha _x + {r_s}_{iy} \alpha _y + {r_s}_{iz} \alpha _z \end{aligned}$$

where ${o_s}_i$ represents the observed rotation angle, rotation rate, or angular acceleration ($a_0$, $a_1$ or $a_2$ from Eq. (7)). The subscript $s=1,2,3$ represents the star tracker identification number (*i.e.,* referring to the camera head unit, where always two out of three are available). The subscript $i=x_{srf},y_{srf}$ denotes the precisely observed values around the two sensitive star tracker axes. The elements of the rotation or mounting matrix between the SRF and GRF are represented by ${r_s}_{ik},~k=x,y,z$. The estimated parameters in the GRF (either angle, rotation rate or angular acceleration) are given by $\alpha _\mathrm{k},~k=x,y,z$.

In fact, the estimated angular rotation rates and accelerations represent averaged values over a moving time window. The averaging method as described in Visser (2008) is used. Please note that in Visser (2008) only one camera head unit was used, but it was already mentioned that a combination of multiple camera would be possible when working with real data.

## Results

The method outlined in this paper relies to a great extent on the reconstruction of the angular rates and accelerations from the star tracker observations. A representative day is taken to assess the quality of this reconstruction by comparison with convenient combinations of the accelerometer observations (Sect. 4.1). The accelerometer calibration estimation results are addressed in Sect. 4.2.

**Fig. 2:**
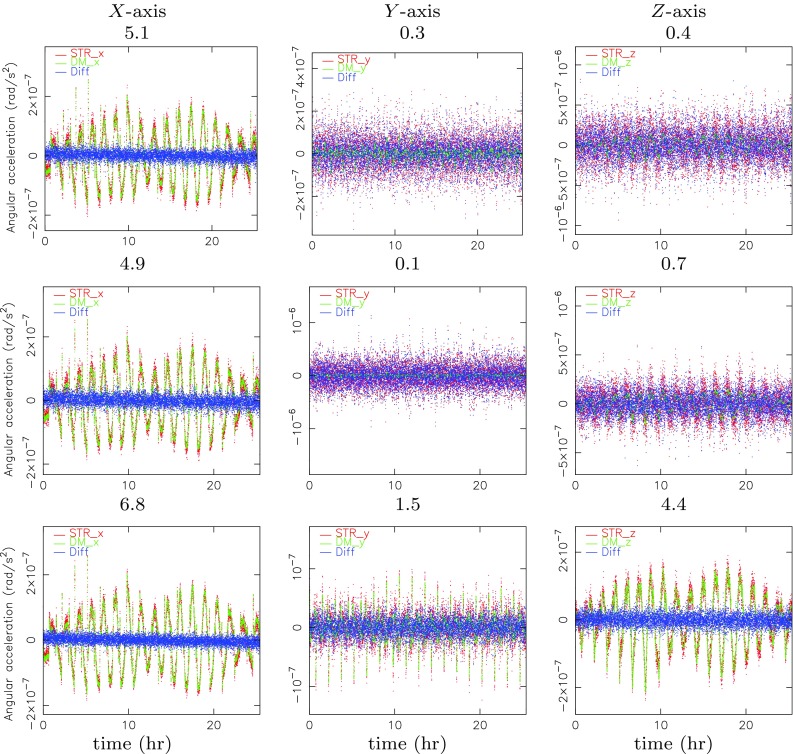
Angular accelerations in GRF derived from the star tracker level-1b product STR$\_$QUA (top row), level-1b STR$\_$QUB (middle row) and combination of STR$\_$QUA/QUB level-1b products (bottom row) versus those derived from the differential gradiometer observations for November 14, 2009. The SNR is indicated above each plot as well. The window used for deriving the angular accelerations is 50 s. Please note that the level-1b products STR$\_$QUA and STR$\_$QUB represent the latest release of the two time series of star tracker quaternions made available by ESA (status January 2017)

### Angular rate and acceleration reconstruction

The angular accelerations that are derived with the method outlined in Sect. 3.2 from star tracker observations can be compared with angular accelerations derived directly from special combinations of the accelerometer observations (Cesare and Sechi 2004):

9 $$\begin{aligned} \dot{\omega }_x ~= & {} ~ -\frac{ {a}_{\mathrm {obs},3,y} - {a}_{\mathrm {obs},6,y} }{2 L_z} +\frac{ {a}_{\mathrm {obs},2,z} - {a}_{\mathrm {obs},5,z} }{2 L_y} \nonumber \\ \dot{\omega }_y ~= & {} ~ -\frac{ {a}_{\mathrm {obs},1,z} - {a}_{\mathrm {obs},4,z} }{2 L_x} +\frac{ {a}_{\mathrm {obs},3,x} - {a}_{\mathrm {obs},6,x} }{2 L_z}\\ \dot{\omega }_z ~= & {} ~ -\frac{ {a}_{\mathrm {obs},2,x} - {a}_{\mathrm {obs},5,x} }{2 L_y} +\frac{ {a}_{\mathrm {obs},1,y} - {a}_{\mathrm {obs},4,y} }{2 L_x}\nonumber \end{aligned}$$

where ${a}_{\mathrm {obs},i,j}~(i=1,\ldots ,6,j=x,y,z)$ represents the observed acceleration for accelerometer *i* and GRF axis *j*. By using the combination of observations by two star camera head units (Sect. 3.2), the consistency with the angular accelerations derived directly from the accelerometers (Eq. (9)) improves significantly. This is reflected by Fig. 2, which displays time series for a typical day (November 14, 2009) of angular accelerations derived from the star tracker quaternions and derived from the accelerometer observations. It can be observed that the signal-to-noise ratio (SNR) improves significantly for 50-s averaged angular accelerations when combining the observations from two star camera head units: from 5.1/4.9 to 6.8, from 0.3/0.1 to 1.5, and from 0.4/0.7 to 4.4, for the GRF *X*, *Y* and *Z* axis, respectively. The SNR is defined as the ratio between the Root-Mean-Square (RMS) of the angular accelerations derived from the very precise accelerometer observations (because of their high precision referred to as signal) and the RMS of the difference of these angular accelerations with the more noisy ones from the star tracker quaternions. It can be observed that for the *Y* axis the SNR is relatively low, which is due to the lower signal for this axis.

**Table 1:** Estimated mean and RMS-about-mean values for the accelerometer bias and drift values, relative to accelerometer 1, and the scale factors obtained from the star tracker observations

	*nr*	*X*-axis	*nr*	*Y*-axis	*nr*	*Z*-axis
Bias (nm/s$^2$)						
Acc. 2	248	− 328.94 ± 3.04	254	12939.45 ± 226.07	254	− 21267.65 ± 746.46
Acc. 3	246	− 301.41 ± 2.49	254	− 9526.68 ± 364.70	251	− 89.47 ± 5.16
Acc. 4	254	− 279.24 ± 3.24	254	3312.12 ± 1303.77	254	15.34 ± 1.76
Acc. 5	248	− 321.47 ± 1.80	254	13479.95 ± 294.43	254	− 10121.48 ± 635.26
Acc. 6	246	− 285.79 ± 2.57	254	9438.67 ± 180.87	245	24.46 ± 3.82
Drift (nm/s$^2$/day)						
Acc. 2	253	− 0.038 ± 0.324	247	− 0.224 ± 0.389	252	− 2.139 ± 0.522
Acc. 3	254	0.007 ± 0.273	248	0.780 ± 0.551	253	0.000 ± 0.267
Acc. 4	245	0.017 ± 0.020	253	3.442 ± 1.003	253	− 0.036 ± 0.534
Acc. 5	253	0.059 ± 0.321	249	− 0.573 ± 0.469	254	3.114 ± 2.726
Acc. 6	253	0.024 ± 0.270	249	− 0.195 ± 0.492	252	− 0.018 ± 0.263
Scale factor						
Acc. 1	230	1.000 ± 0.001	254	0.996 ± 0.016	246	1.000 ± 0.004
Acc. 2	241	0.993 ± 0.003	254	0.996 ± 0.017	251	1.003 ± 0.004
Acc. 3	252	0.996 ± 0.015	254	0.995 ± 0.016	251	0.998 ± 0.005
Acc. 4	227	0.999 ± 0.001	254	0.996 ± 0.017	248	0.997 ± 0.007
Acc. 5	254	0.991 ± 0.003	254	0.996 ± 0.017	246	0.999 ± 0.003
Acc. 6	253	0.994 ± 0.015	254	0.997 ± 0.017	244	0.999 ± 0.004

Use was made of 254 daily arcs, where a 3$\sigma$ editing was applied (number of used arcs indicated by *nr*)

The improvement of the SNR when combining the pairs of precisely observed orientation angles for two star trackers is in accordance with expectation. For November 14, 2009, star tracker A (top row in Fig. 2) has identification number 2 and star tracker B (middle row in Fig. 2) has identification number 1. When looking at Fig. 8.4 in HPF (2014), it can be derived that the worst observed orientation angle for star tracker 1 is predominantly around the GRF *Y* axis and for star tracker 2 around the GRF *Z* axis. When combining the observations of the two star trackers, indeed the largest improvements are obtained for the GRF *Y* and *Z* axes.

The choice of selecting a 50-s averaging window was a trade-off between minimizing model error and reducing the impact of star tracker observation noise (not shown here). Although the 50-s averaging is applied to the accelerometer observations and the derived angular velocities and accelerations, this does not lead to a perfect 50-s averaging of all elements in the observation equations. For example, the square of the 50-s averaged value for $\omega _x$ is not perfectly identical to the 50-s averaged value for the square of $\omega _x$ (${\omega _x}^2$ is one of the elements of the matrix $\mathbf {R}$ in Eq. (6)):

10 $$\begin{aligned} \mu ({\omega _x}^2) \ne \left[ \mu (\omega _x) \right] ^2 \end{aligned}$$

where $\mu$ represents the 50-s averaging.

With each derivative in time, the star tracker noise is amplified. It was therefore found that the largest uncertainties apply to the estimated angular accelerations using the methodology outlined in Sect. 3.2. The associated unweighted least-squares estimation process allows to obtain unweighted formal errors for these estimated angular accelerations. These formal errors were scaled by the RMS-of-fit of the yaw, pitch and roll angles derived from the star tracker quaternions. The RMS-of-fit is the RMS of the differences between these angles and the associated values of the fitted polynomials (Eq. (7)). For a 50-s time interval, it was found that the RMS of the ratio of estimated angular accelerations and formal errors is significantly above 3 (typically between 4 and 9, thus much better than 99.7% confidence interval assuming errors with Gaussian distribution). For smaller time intervals, *e.g.,* 40 s, this ratio is regularly below 3.

### Calibration parameters

The estimation of the accelerometer calibration parameters is done in daily batches. As outlined in Sect. 2, 254 days were selected where the same pair of camera head units was observing for the entire day. In total, 48 parameters are estimated for each daily arc consisting of 15 accelerometer biases and 15 bias drifts, all relative to accelerometer 1 (Fig. 1), and 18 scale factors (6 accelerometers $\times$ 3 GRF axes). It has to be noted that in fact the products of the accelerometer bias and bias drifts with the associated scale factors are estimated. When interpreting the bias and bias drift values, this has then of course to be taken into account. For scale factors equal to 1, the interpretation would then be straightforward.

The parameters were estimated by using the method of unweighted least-squares to solve the observation equations (Eq. (1)). Two different implementations were used, where for the first implementation the full accelerometer observations were used and for the second implementation these observations were reduced first by their daily mean, and this mean was added afterward. The second implementation can be considered as a remove-restore method. It was found that both implementations led to identical parameter values, which is to be expected in case of a stable estimation problem. However, the remove-restore method is conceptually numerically more stable as will be shown in “Appendix B.” For the latter implementation, only high correlations remain between the scale factors of the accelerometer *Y* axes.

An a priori gravity field model is required to compute the gravity gradients as part of the observation equations (Eq. (1)). To this aim, the EIGEN5C gravity field model was selected (Foerste et al. 2008). It was shown in Visser (2008) that the method used in this paper is not very sensitive to errors in the gravity field model, for example no difference between estimated accelerometer scale factors could be observed for the end-to-end simulated data when using the much older EGM96 (Lemoine 1997) and JGM2 (Tapley et al. 1996) gravity field models. As an extra verification of the implemented software and methodology, also Eq. (11) from Visser (2008) was used to estimate the arm lengths with the assumption that the scale factors are equal to 1. The estimates for the arm lengths vary between 48 and 51 cm, *i.e.,* in general within a few percent of the actual arm lengths of 50.0 and 51.4 cm. This is comparable to the variation of estimated scale factors, which can be up to 0.017 for especially the *Y* axes (Table 1).

**Table 2:** Comparison between accelerometer bias and drift values, relative to accelerometer 1, obtained from the star tracker observations and those by precise orbit determination taken from Visser et al. (2016)

	Bias (nm/s$^2$)			Drift (nm/s$^2$/day)		
	*X*-axis	*Y*-axis	*Z*-axis	*X*-axis	*Y*-axis	*Z*-axis
From star tracker observations						
Acc. 2	− 332.4146	13,078.5800	− 20,307.6890	0.0073	$-$ 0.1828	$-$ 1.8468
Acc. 3	− 304.2294	− 9936.3365	− 88.9736	0.0061	0.7485	0.0030
Acc. 4	− 282.8832	1778.9331	17.0539	0.0075	3.2176	$-$ 0.0039
Acc. 5	− 319.9281	13,729.3195	− 10,824.0599	$-$ 0.0035	$-$ 0.4030	1.4348
Acc. 6	− 288.3063	9576.0872	24.0190	0.0057	$-$ 0.1998	$-$ 0.0006
From precise orbit determination (Visser et al. 2016)						
Acc. 2	− 330.3978	13,075.7939	− 20,345.9043	0.0067	$-$ 0.1823	$-$ 1.8018
Acc. 3	− 303.9239	− 9921.2734	− 94.9370	0.0052	0.7224	0.0061
Acc. 4	− 282.1677	1848.7939	5.2041	0.0065	3.1275	$-$ 0.0010
Acc. 5	− 321.4763	13,712.3486	− 10,646.8096	$-$ 0.0036	$-$ 0.3852	1.1874
Acc. 6	− 287.5175	9588.0723	18.3239	0.0051	$-$ 0.2157	0.0028

The accelerometer scale factors were taken equal to 1

Table 1 contains results of the estimation of accelerometer calibration parameters with the method outlined in Sect. 3. A 3$\sigma$ editing was applied, where parameter values that deviate more than 3 times the RMS-about-mean were eliminated. In general, less than a few percent of the values were eliminated due to this editing. The largest number of edited values occurred for the *X*-axis scale factors of accelerometers 1 and 4: up to 11%. However, this is due to the very low RMS-about-mean value for these scale factors and it was found that the mean value of all used scale factor values in Table 1 changed by less than 0.001 when no editing was applied.

It can be observed that the averaged scale factors are very close to 1 for all accelerometer axes: the deviation from 1 is always smaller than 0.01. The RMS-about-mean values are smaller than 0.01 for all accelerometer axes, except for all the *Y* axes and for the *X* axes of accelerometers 3 and 6 for which the RMS-about-mean is still smaller than 0.02. Please note that for the *Y* axes, the formal errors are the largest (Table 3 in “Appendix B”) which is consistent with the larger RMS-about-mean. In addition, the formal errors for the *X* axis scale factor for accelerometers 3 and 6 are relatively large as well, although it is larger for accelerometer 4 for which the performance is very good (RMS-about-mean equal to 0.001). However, for the accelerometers 3 and 6 the term $\dot{\omega }_ y$ is included (Eq. (6)), which is determined relatively badly (Fig. (2)). Together with the low signal due to the drag-free control, the larger values for the RMS-about-mean can be explained. It can be observed that all scale factors for the accelerometer *Y* axes are a little bit smaller than 1. This can probably be attributed to the earlier mentioned small model error due to the averaging interval 50 s. The used implementations of the method do show, however, that the scale factors are consistent at a level which is significantly better than 0.01. The fact that the estimated scale factors are close to 1 is a verification of not only the high quality of the calibration of the level-1b accelerometer data, but also of the high quality of the star tracker quaternion data.

Table 1 also includes the mean values for the daily accelerometer bias and bias drifts together with their RMS-about-mean. The latter values are not representative for the precision of the bias estimates since the associated time series were not reduced for the systematic bias drifts. The latter are very big for certain accelerometer axes (up to more than 3 nm/s$^2$/day). The RMS-about-mean values do, however, show the difference in behavior between sensitive and less-sensitive axes: the values are big for all *Y* accelerometer axes, because the less-sensitive axis of accelerometer 1 serves as reference which has significant bias drift (about 0.18 nm/s$^2$/day, *cf.* Figs. 6 and 7 in Visser et al. (2016), please note as well that 4 out of 6 *Y* accelerometer axes are less sensitive). In addition, the RMS-about-mean values are big for the *Z* axes of accelerometers 2 and 5 (see also Fig. 1).

**Fig. 3:**
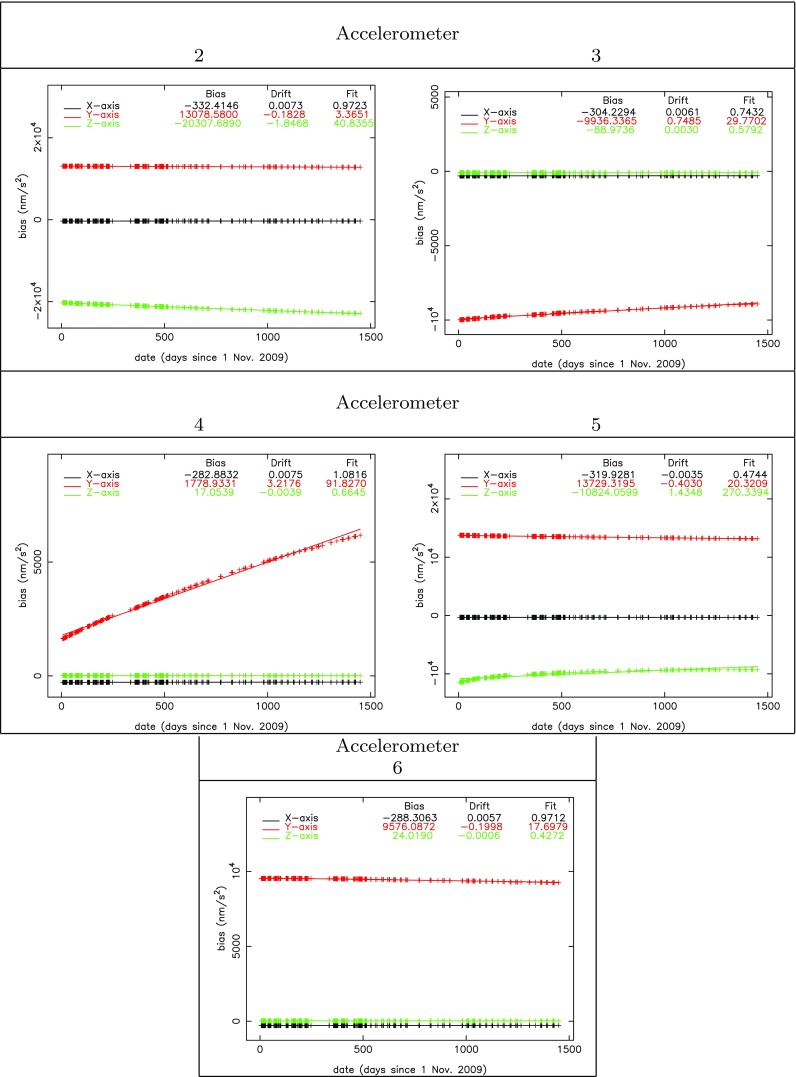
Estimated accelerometer biases (nm/s$^2$) and drifts (nm/s$^2$/day) relative to accelerometer 1 for the *X*, *Y* and *Z* axes. Use was made of 254 daily arcs. The RMS-of-fit for a linear regression is displayed as well (nm/s$^2$), together with the relative bias (epoch November 1, 2009) and drift of the linear regression. The accelerometer scale factors were taken equal to 1

The estimated daily bias drift values are in general not very precise: in most cases the RMS-about-mean values are larger than the associated values for the 254-day mean. The drift of the accelerometer in one day is in general much smaller than 1 nm/s$^2$ (except for a few axes which necessitated the estimation of those drifts). More stable values can be obtained by a linear regression of the 254 daily bias values, just as was also done in Visser et al. (2016). In Section 3 of Visser et al. (2016), it is stated that the scale factors were kept fixed at a value equal to 1. In order to allow a comparison, new values for the accelerometer bias and bias drifts were estimated with the method outlined in this paper where the scale factors were also kept fixed to 1. A linear regression was applied to the new time series as well. The so-obtained bias drift values and bias values at the epoch of November 1, 2009, are included in Table 2 and displayed together with the RMS-of-fit of the linear regression in Fig. 3. The associated values from the precise orbit determinations described in Visser et al. (2016) are included for comparison and validation. The consistency between the accelerometer bias and bias drift values estimated by precise orbit determination in Visser et al. (2016) is very good, where all values are relative to the reference accelerometer 1. For all *X* axes, the bias at epoch is consistent to within 2 nm/s$^2$ and the bias drift value within 0.001 nm/s$^2$/day. For the *Y* and *Z* axes, the bias and bias drift values match in general quite well, where lower consistency levels can be explained by the different behavior of sensitive and less-sensitive axes, and the lower precision of bias estimates by precise orbit determination for these axes (Visser et al. 2016). The accelerometer bias parameters estimated by precise orbit determination are especially precise for the GRF *X* axis and orders of magnitude less precise for the GRF *Z* axis. For the method outlined in this paper, the RMS-of-fit of the linear regression in Fig. 3 is much better for the *Z* axis when only sensitive axes are involved (*cf.* Figs. 6 and 7 in Visser et al. 2016). Possibly the results in this manuscript can be used to enhance the accelerometer bias and bias drift values and support GOCE-based long-wavelength gravity field determination from GPS SST observations and thermospheric density and winds retrieval from the accelerometer observations.

It can be observed in Fig. 3 that the RMS-of-fit of the linear regression is better than 1.09 nm/s$^2$ for all relative biases for the accelerometer *X* axes. This is also the case for all the accelerometer *Z* axes, except for the less-sensitive *Z* axes of accelerometers 2 and 5. For the *Y* axes, the consistency is worse since the less-sensitive axis of accelerometer 1 serves as reference.

## Conclusions

A method for validating scale factors for all six GOCE accelerometers from star tracker quaternions has been applied successfully to real data. In addition, the method provides values for the accelerometer biases and bias drifts with respect to a reference accelerometer (in this case GOCE accelerometer 1). The method was originally implemented and tested with simulated end-to-end simulator data before the launch of GOCE. In the pre-launch implementation, use was made of the star tracker observations of a single star camera head unit leading to a relatively coarse reconstruction of angular rates and accelerations around the bore axis of this unit. The method was enhanced by including a procedure for the attitude motion reconstruction from two time series of two differently oriented camera head units. It has been shown that this procedure leads to a much better consistency with angular accelerations that are derived directly from differential accelerometer observations.

The improved attitude reconstruction products were included in the validation of accelerometer calibration parameters. In total, 254 days were selected that cover the GOCE operational mission period from the beginning in November 2009 to the end in October 2013. The resulting scale factors were found to be very close to 1: the maximum deviation for the 254-day averages is significantly below 0.01 and the associated RMS-about-mean values are below 0.02. The RMS-about-mean values were in general found to be consistent in a relative sense with formal error estimates. When taking the accelerometer scale factors equal to 1, the resulting biases and long-term bias drifts were found to be consistent with the values published in Visser et al. (2016), which are based on precise orbit determinations. It is therefore concluded that it has been successfully demonstrated that in addition to scale factors for all GOCE accelerometer, also significant information about their biases and bias drifts can be extracted from the star tracker quaternions relative to a reference accelerometer.

## Figures and Tables

**Fig. 4:**
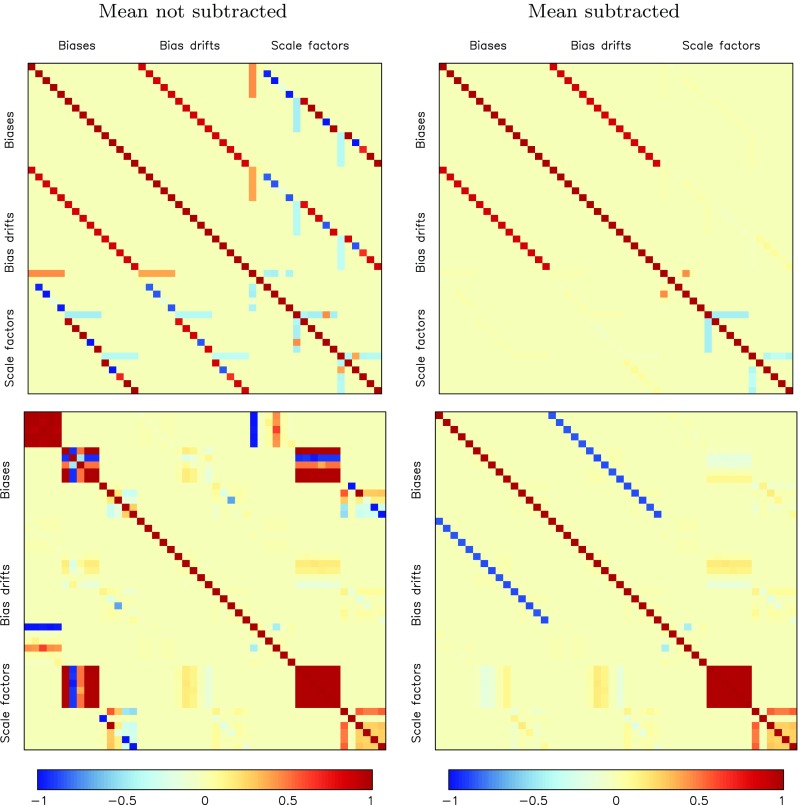
Normal matrices (top) and their inverse (bottom) for estimated accelerometer calibration parameters for November 14, 2009. The plots on the left hold for the case where the accelerometer observations are not reduced by their mean value. The plots on the right hold for the case where they were reduced for their mean. The matrix elements are normalized by the square roots of the diagonal elements. The sequence of estimated parameters from left to right or from top to bottom is as follows: differential biases for, respectively, the GRF *X*, *Y*, and *Z* axis (3 $\times$ 5 = 15 parameters), differential bias drifts for, respectively, the GRF *X*, *Y* and *Z* axes (again 3 $\times$ 5 = 15 parameters) and the scale factors for, respectively, the GRF *X*, *Y* and *Z* axes (3 $\times$ 6 = 18 parameters)

**Fig. 5:**
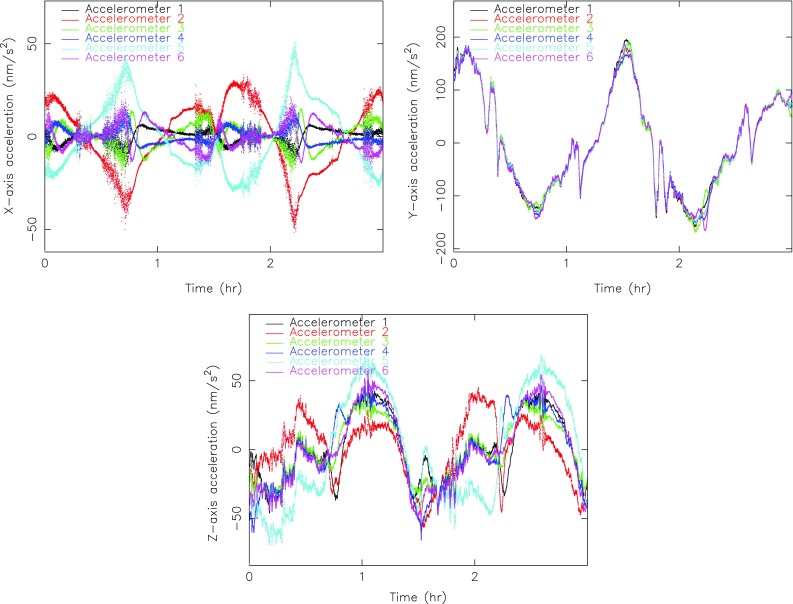
Time series of accelerometer observations for the first three hours of November 14, 2009, for all three GRF axes. The mean of the accelerometer observations has been removed for easy comparison

## Appendix A: Selected days

The 254 selected days for which no change in the selection of two star tracker camera head units occurred are:

**Table:**

2009								
091107	091109	091111	091112	091113	091114	091115	091116	091117
091118	091119	091120	091121	091122	091123	091125	091128	091209
091211	091212	091213	091215	091216	091217	091218	091219	091220
091221	091222	091223	091224					
2010								
100107	100109	100113	100114	100115	100116	100117	100118	100120
100121	100123	100124	100129	100130	100131	100205	100206	100207
100209	100210	100307	100309	100310	100311	100312	100313	100314
100315	100316	100317	100318	100403	100405	100407	100411	100412
100413	100415	100416	100417	100418	100419	100421	100422	100424
100501	100508	100509	100510	100511	100512	100513	100514	100515
100516	100517	100518	100519	100520	100524	100606	100607	100608
100609	100610	100611	100612	100613	100614	100615	100616	100618
100619	100623	100706	101002	101003	101023	101024	101027	101030
101031	101103	101104	101105	101106	101107	101108	101109	101110
101114	101130	101201	101202	101203	101204	101205	101209	101210
101211	101212	101213	101215	101218	101225	101229	101230	
2011								
110129	110130	110202	110203	110204	110205	110206	110212	110218
110219	110223	110224	110226	110227	110228	110301	110302	110303
110304	110305	110306	110307	110308	110309	110310	110311	110312
110324	110327	110328	110329	110330	110331	110401	110402	110422
110424	110514	110522	110613	110620	110623	110712	110720	110722
110723	110810	110815	110816	110820	110821	110913	110914	110919
111008	111013	111017	111212	111215				
2012								
120204	120208	120209	120313	120403	120411	120413	120509	120511
120708	120710	120711	120728	120803	120807	120809	120810	120826
120901	120902	120903	120907	120909	121001	121002	121007	121031
121105	121130	121201	121204	121229	121230			
2013								
130123	130128	130129	130226	130227	130303	130324	130331	130403
130420	130531	130601	130629	130724	130728	130730	130822	130823
130827	130921	130922	130926	130928	131020			

where each day is indicated by *YYMMDD*, with *YY* indicating the year after 2000, *MM* indicating the month, and *DD* the day of the month.

## Appendix B: Covariance analysis

As mentioned in Sect. 4, two different implementations were used for solving the observation equations. It is interesting to analyze the numerical stability of these two implementations. It can be anticipated that very high correlations arise between bias parameters if the accelerometer observations suffer from very large biases compared to the variance of the signals observed by the accelerometers. This is reflected by Fig. 4, which shows the normalized normal matrices and their inverse (or covariance matrix) for the two implementations for a typical day (November 14, 2009). The matrices were normalized for this Appendix in order to improve visualization of their structure. Normalized means that the matrix elements are scaled by the square roots of the diagonal elements:

11 $$\begin{aligned} \tilde{N}_{ij} = \frac{N_{ij}}{\sqrt{N_{ii}}\sqrt{N_{jj}}} \end{aligned}$$

where $\tilde{N}_{ij}$ represents the normalized matrix element $N_{ij}$ for row *i* and column *j*. For the first implementation (left in Fig. 4), it can be observed that indeed very high correlations (*i.e.,* very close to 1) arise between the bias parameters for especially GRF *X* and *Y* axes, which can be explained by the predominantly identical bias values for all accelerometer *X* axes and very large bias values for all accelerometer *Y* axes (Table 1). These correlations disappear for the second remove-restore implementation (right in Fig. 4). The condition number (ratio of the highest and lowest eigenvalue) for the normalized matrix is equal to $3.2 \times 10^7$ for the first implementation and $7.2 \times 10^2$ for the second implementation, showing indeed the better conditioning for the remove-restore method.

Very high correlations remain, also for the remove-restore implementation, between all scale factors for the *Y* accelerometer axes. The correlations are smaller for the *Z* accelerometer axes and close to zero for the *X* accelerometer axes. The latter can be explained by the drag-free control which leaves a very small non-gravitational signal in the *X* direction. For the *Y* and *Z* axes, significant non-gravitational signal is left which is observed by all accelerometers (of course in addition to the rotational terms) leading to high(er) correlations in the covariance matrix. This is corroborated by Fig. 5, which indeed shows that the observed accelerations are very similar for all *Y* axes for representative time series.

**Table 3:** Formal errors for the estimated calibration parameters for November 14, 2014

	*X*-axis	*Y*-axis	*Z*-axis	*X*-axis	*Y*-axis	*Z*-axis
Bias (nm/s$^2$)						
Acc. 2	0.9909	19.1515	15.2943	0.0473	0.0474	0.0479
Acc. 3	0.9985	16.0440	1.1662	0.0472	0.0480	0.0481
Acc. 4	1.1451	3.4890	0.0530	0.0472	0.0477	0.0478
Acc. 5	0.9910	21.2497	5.0191	0.0473	0.0474	0.0475
Acc. 6	0.9973	14.2458	0.9991	0.0472	0.0476	0.0477
Drift (nm/s$^2$/day)						
Acc. 2	0.0771	0.0774	0.0785	0.0771	0.0774	0.0785
Acc. 3	0.0771	0.0788	0.0789	0.0771	0.0788	0.0789
Acc. 4	0.0771	0.0782	0.0782	0.0771	0.0782	0.0782
Acc. 5	0.0771	0.0773	0.0776	0.0771	0.0773	0.0776
Acc. 6	0.0771	0.0779	0.0781	0.0771	0.0779	0.0781
Scale factor						
Acc. 1	0.0030	0.0015	0.0007	0.0030	0.0015	0.0007
Acc. 2	0.0007	0.0016	0.0008	0.0007	0.0016	0.0008
Acc. 3	0.0038	0.0015	0.0013	0.0038	0.0015	0.0013
Acc. 4	0.0064	0.0016	0.0010	0.0064	0.0016	0.0010
Acc. 5	0.0006	0.0015	0.0004	0.0006	0.0015	0.0004
Acc. 6	0.0038	0.0016	0.0010	0.0038	0.0016	0.0010

A uniform weight of 1 nm/s$^2$ for the accelerometer observations is applied. The values on the left hold for the case where the accelerometer observations are not reduced by their mean value. The values on the right hold for the case where the mean has been subtracted

The better numerical stability with the remove-restore implementation is also reflected by the formal errors derived from the scaled inverse of the normal matrix. A—rather arbitrary—scaling is applied to serve as example. A Gaussian noise level for the accelerometers equal to 1 nm/s$^2$ was adopted. This led to the formal errors included in Table 3. The values in this table confirm that the remove-restore implementation leads to much smaller formal errors for the bias parameters, but does not affect the formal errors of the bias drifts and scale factors. The latter is consistent with the expectation that in case of (a sufficiently) stable estimation process, the remove-restore method only leads to smaller values for the estimated biases (*i.e.,* before the restore step) and not for the bias drifts and scale factors. Of course the bias values become the same as well after adding the originally removed mean values of the accelerometer observations.
